# The Landscape on Access to Maternal and Child Health Services During the COVID‐19 Pandemic in South Africa: A Scoping Review

**DOI:** 10.1155/ipid/9065224

**Published:** 2026-01-29

**Authors:** Delia Chikuse, Abebe Sorsa Badacho, Jeannine Uwimana-Nicol, Lynn Hendricks, Juliet Charity Yauka Nyasulu

**Affiliations:** ^1^ Department of Global Health, Faculty of Medicine and Health Sciences, Division Health Systems and Public Health, Stellenbosch University, Cape Town, South Africa, sun.ac.za; ^2^ School of Public Health, College of Health Sciences and Medicine, Wolaita Sodo University, Wolaita Sodo, Ethiopia, wsu.edu.et; ^3^ Department of Global Health, Faculty of Medicine and Health Sciences, Division of Epidemiology and Biostatistics, Centre of Evidence-Based HealthCare, Stellenbosch University, Cape Town, South Africa, sun.ac.za; ^4^ School of Public Health, College of Medicine and Health Sciences, University of Rwanda, Kigali, Rwanda, ur.ac.rw; ^5^ Health Systems Strengthening, AFRIQUIP, Johannesburg, South Africa

**Keywords:** child∗ health, children, COVID-19, effects of COVID-19, low- and middle-income countries (LMICs), maternal and child health services, maternal health, women, “South Africa.”

## Abstract

**Background:**

In early March 2020, the World Health Organization (WHO) declared COVID‐19 a pandemic. In South Africa, the first case was confirmed in early March 2020. According to the WHO, disruptions in essential services due to the COVID‐19 pandemic occurred worldwide. The COVID‐19 pandemic affected access to maternal and child health (MCH) services in many countries, including South Africa. The study aimed to map and describe the existing evidence on the impact of the COVID‐19 pandemic on the access to and delivery of maternal, neonatal, and child health (MNCH) services in South Africa.

**Methodology:**

This was a scoping review of studies published between 2020 and 2023. We searched databases such as PubMed, MEDLINE, EBSCOhost, and Google Scholar. Data were exported to the Rayyan software, where screening, checking of duplicates, and selection of final studies for review were performed. The information from the identified studies was exported to ATLAS.ti 23.1 software for analysis. Content analysis was performed, and data were presented in predetermined themes using the MCH cascade.

**Results:**

The results from 25 articles showed a mixed view, whereby some studies showed a decrease at the beginning of the pandemic in April 2020, in the uptake of family planning, antenatal care, labor and delivery, postnatal care, under‐five immunizations, and cervical cancer screening services. However, other studies found increased uptake of family planning, antenatal care, labor and delivery, and under‐five immunization services. Some studies showed resilience in the overall first antenatal visits, adolescents’ visits to family planning, and postnatal care, as they remained constant.

**Conclusion:**

The findings show both positive and negative impacts of the COVID‐19 pandemic on MNCH services in South Africa. While the pandemic significantly disrupted access to essential services, some areas demonstrated resilience, with increased visits for antenatal care, adolescent family planning, and postnatal services. These insights are critical for guiding decision‐makers, health managers, and frontline healthcare workers in preparing for future public health emergencies. Ensuring continuity of MNCH services during crises must be a priority. Strengthening the health system and building resilience are essential to safeguard MCH, even in the face of disruptions.

## 1. Introduction

Coronavirus disease 2019 (COVID‐19) is defined as “an illness caused by a novel coronavirus called Severe acute respiratory syndrome coronavirus 2 (SARS‐CoV‐2) (formerly called 2019‐nCOV)” [1]. According to Jin et al. [2], COVID‐19 was identified in Wuhan City, China, in December 2019. In early March 2020, the World Health Organization (WHO) declared COVID‐19 a pandemic [3]. The first case in South Africa (SA) was confirmed in early March 2020 [4]. In the first quarter of 2023, SA recorded the highest cumulative COVID‐19 cases on the continent with 5,050,050 cases [5]. The COVID‐19 pandemic affected access to maternal and child health (MCH) services in many countries, including SA. The MCH services cascade covers the well‐being of women, newborns, and children through the care they receive in adolescence, pregnancy, delivery, postnatal, and childhood [6]. These include access to family planning, antenatal care (ANC), labor and delivery, postnatal care, and immunizations [6]. Sines et al. [6] further indicate that if mothers and children are given quality care, millions of lives would be saved. Family planning (FP) refers to services provided to individuals or couples to ensure they have information on planning and spacing pregnancy [7]. ANC monitors the health of a pregnant woman and the baby to be born so that complications can be detected and treated early [6–8]. Labor and delivery services ensure that skilled birth attendants attend to women in a clean environment and have a safe delivery [6, 7]. Postnatal care “aims at preventing and managing complications that may endanger the survival of the mother and baby” [9]. Immunizations protect children against diseases from birth and aim to reduce the impact of vaccine‐preventable diseases by achieving high immunization coverage rates in the community [10]. These services were affected as the COVID‐19 pandemic disrupted them.

According to WHO [11], disruptions in essential services due to COVID‐19 occurred worldwide, affecting reproductive, maternal, neonatal, and child health (RMNCH) and nutrition. In countries such as Haiti and Sierra Leone, Aranda et al. [12] observed a decline in ANC visits. Aranda et al. [12] further observed that facility‐based delivery decreased in Haiti, Liberia, Mexico, and Sierra Leone. The decline in ANC and facility‐based deliveries was attributed to fear of contracting COVID‐19 and lockdown regulations [12]. Burger and Mchenga [13] also indicate that in SA, fear of contracting coronavirus during the COVID‐19 pandemic was one of the top reasons people did not seek care.

In addition, restrictions in movement, access to transport, and financial problems were cited as other factors that hindered access to health facilities [11, 14]. Similarly, Mochache et al. [15] indicated that when facilities were being decontaminated and healthcare workers (HCWs) were sick with COVID‐19, service deliveries were affected as facilities were closed. It could be argued that this affected service delivery, as in Pakistan, it is reported that there was a gradual and consistent decline in the utilization of all RMNCH services, whereby FP decreased by 50%, antenatal first visits decreased by 37.5%, and postnatal visits decreased by 37.5% during peak COVID‐19 waves [16].

Immunization coverage within and across countries declined to 81%, as shown by children who accessed 3 doses of diphtheria, pertussis, and tetanus (DPT3) [10]. Factors such as misinformation, service and supply chains, and containment measures during COVID‐19 contributed to challenges in accessing and availability of immunizations [10]. In SA, utilization of RMNCH services such as ANC, family planning, and immunizations declined due to lockdown measures and a lack of medical supplies for HCWs, whereas ANC visits and hospital‐based deliveries decreased by 17.5% and 28.8%, respectively [17, 18]. Even though much has been documented on how the MCH services were affected, not much has been written on the landscape of these effects in Africa. Therefore, this study aimed to map and describe the existing evidence on the impact of the COVID‐19 pandemic on the access to and delivery of MCH services in SA.

## 2. Methodology

### 2.1. Study Design

We conducted a scoping review with a defined search strategy and selection criteria for publications to be included as per the developed protocol, which is included as Supporting Information (available here). The scoping review was reported using the Preferred Reporting Items for Systematic Reviews and Meta‐Analyses Extension for Scoping Review (PRISMA‐SCR) guidelines [19] (refer to File 1).

Aim: This study aimed to map and describe the existing evidence on the impact of the COVID‐19 pandemic on the access to and delivery of MCH services in SA. The research protocol of the scoping review is included in the Appendix (Appendix 1).

### 2.2. Search Strategy

We searched for publications from January 2020 to September 2023 from the following databases: PubMed, MEDLINE, EBSCOhost CINAHL, PROSPERO, Scopus, Google Scholar, Google, and JBI. In addition, we searched for gray literature from websites such as CDC Africa, Database of Abstract Reviews of Effect, USAID, UNICEF, National Department of Health (NDoH), Save the Children, Health Systems Trust, SA Medical Research Council (SAMRC), Human Sciences Research Council (HSRC), WHO Africa Region, and the WHO International Conference. The references from the included articles were used for the final search.

The search strategy combined MeSH terms with Boolean operators as shown in Table 1. The following key terms were used: effects of COVID‐19, women, children, maternal health, child∗ health, access, maternal health services, MCH, COVID‐19, “South Africa,” and Low‐ and Middle‐IncomeCountries (LMICs).

**Table 1:** MeSH terms and Boolean operators used for searching articles.

1. (‘Effects COVID‐19’ [title/abstract] OR ‘Impact COVID‐19’ [title/abstract]) OR (effects COVID‐19 OR impact COVID‐19 [MeSH terms])
2. (Women [title/abstract] OR female [title/abstract] OR woman [title/abstract]) OR (women [MeSH terms])
5. (Infant [title/abstract] OR preschool minors [title/abstract] OR ‘under‐five years old child∗’ [title/abstract]) OR (children OR preschool minors OR under‐five children [mesh terms])
6. (‘Maternal health’ [title/abstract] OR ‘Women’s health’ [title/abstract]) OR (maternal health OR women’s health [MeSH terms])
7. (‘Child∗ health’ [title/abstract] OR health, child∗ [title/abstract] OR children’s health [title/abstract] OR health, children’s [title/abstract]) OR (child health [MeSH terms])
8. (MCH [title/abstract] OR MNCH [title/abstract] OR ‘maternal health services’ [title/abstract] OR ‘child health services’ [title/abstract] OR ‘maternal‐ child health
9. (‘South Africa’ [title/abstract]) OR (South Africa [MeSH terms])
10. (LMIC [title/abstract] OR ‘lower middle‐income countries’ [title/abstract]) OR (LMIC OR lower middle‐income countries [MeSH terms])
11. Access, maternal health services [title/abstract]
12. (COVID‐19 [title/abstract] OR SARS‐COV‐2 [title/abstract] OR COVID‐19 [title/abstract] OR coronavirus [title/abstract]) OR COVID‐19 OR COVID‐19 OR SARS‐COV‐2 OR COVID‐19 OR coronavirus [MeSH terms])
13. (Pandemic [title/abstract]) OR (pandemic [MeSH terms])
14. #1 AND #12 AND #13 S12
15. #6 AND #7 AND #8 AND #11 13
16. #6 AND #8 AND #11S 15
17. #9 OR #10
18. #14 AND #16 AND #17
19. #2 AND #5 AND #6 AND #14 AND #16
20. #19 AND # 16 AND #17
21. #2 AND #5 AND #16 AND #10
22. #2 AND #5 AND #6 AND #16 AND #9

*Note:* Child^∗^ = child/children.

### 2.3. Selection Criteria

Articles were included in the study if they focused on the impact of COVID‐19 on access to MCH services in SA for women and under‐five children. Articles that did not focus on the impact of COVID‐19 on access to MCH services in SA for women and under‐five children were excluded. Articles that were multinational and included SA were included. The specific MCH services of focus were on family planning, ANC, labor and delivery, postnatal care, cancer screening, and under‐five immunizations. The articles were included if they were published between January 2020 and September 2023 and English language publication. The articles included both primary and secondary studies, commentaries, and gray literature.

### 2.4. Screening Process

Identifying duplicates and screening for identified articles were performed using Rayyan software to facilitate initial title and abstract screening [20]. Independent double screening of the title and abstract and full‐text review was performed by the authors, Delia Chikuse and SN. In terms of conflicts, discussions were held until a consensus was reached, and where there was no resolution to the conflict, a senior author (Juliet Charity Yauka Nyasulu) was reached to resolve the dispute. The author (Delia Chikuse) further searched the gray literature and the article references of each included study, screened them, and identified relevant articles that were included. Critical appraisal was not performed because the review aimed to identify the available evidence and not to assess the quality of the available evidence in the articles.

### 2.5. Data Analysis

Data were analyzed through content analysis, which included both qualitative and quantitative studies. We used predetermined themes according to the mother–baby service package cascade, which included family planning, ANC, labor and delivery, postnatal care, cervical cancer screening, and under‐five immunization [19]. A qualitative data analysis software ATLAS.ti was used for organizing the data analysis. The codes were generated according to the predetermined themes, as shown in Table 2.

## 3. Results

**Figure 1:**
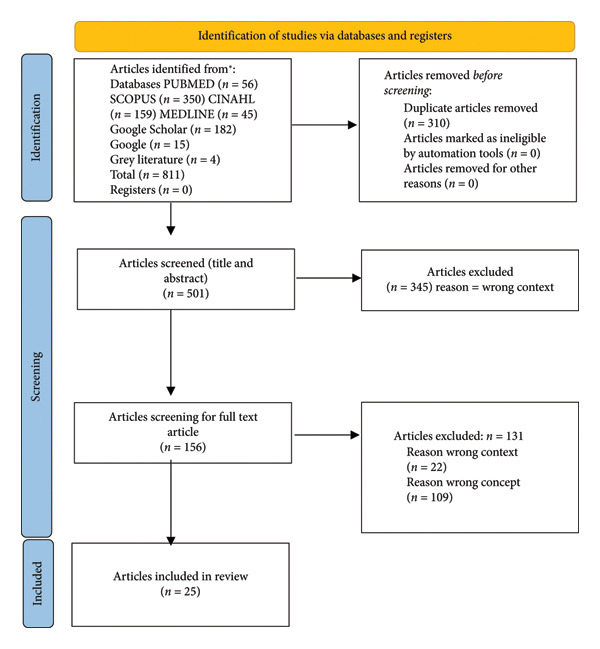
PRISMA‐SCR flow diagram. Source: Page MJ, et al. BMJ 2021; 372: n 71. doi: 10.1136/bmj.n71. This work is licensed under CC BY 4.0. To view a copy of this license, visit https://creativecommons.org/licenses/by/4.0/.

The database search yielded 811 articles, of which 807 articles were from databases and 4 articles were identified through further gray literature search and article reference search. 310 duplicates were removed from the database search, and 501 articles were screened for titles and abstracts. 345 articles were excluded as they did not meet the inclusion criteria. 156 articles remained for full‐text article screening from the initial search results. After full text review, 131 articles were removed for the following reasons: *n* = 22 wrong context and *n* = 109 wrong concept. The process is presented using the PRISMA‐SCR flow diagram as shown in Figure 1.

### 3.1. Description of the Included Studies

A total of 25 articles that met the inclusion criteria of the scoping review were included for final review. All the 25 studies and articles that were included were published between January 2020 and September 2023.

Ten of the 25 studies were performed in high‐income countries (HICs), LMICs, and Sub‐Saharan African regions, in which SA was one of the study countries. The remaining 15 studies were specifically performed in SA only. The articles included scoping reviews, systematic scoping reviews, and/or systematic reviews, qualitative studies, quantitative and participatory studies, cross‐sectional surveys, literature reviews, reports, commentary, correspondences, and descriptive data analysis. The studies focused on adolescents aged 10–19 years (*n* = 2), women, pregnant, postnatal mothers, children and babies (*n* = 18), adults and the general population (*n* = 1), and HCWs (*n* = 4).

### 3.2. The Available Evidence

The available eveidenceThe results were analyzed based on the predetermined themes about access and delivery of MCH services using the maternal and neonatal child health (MNCH) cascade: family planning, ANC, labor and delivery, postnatal care, and under‐five immunizations.

Evidence shows that 19/25 (76%) of the analyzed papers reported a decline in the use of MCH health services utilization during the COVID‐19 pandemic. Of those, eight focused on family planning, six on ANC, five on labor and delivery, two on postnatal care, and nine on immunizations for children under five.

Four ANC articles, three labor and delivery articles, two vaccination articles, and five FP articles were among the 12 articles that discussed the rise in the use of health services. Fifteen articles reported on changes in service utilization, of which five reported on family planning, five on ANC, two on labor and delivery, three on postnatal care, and three on immunization. Two studies reported on the socioeconomic impact of the COVID‐19 pandemic.

On the other hand, 15 articles reported on the delivery of MNCH services: one article reported on the general impact of MNCH service delivery, five articles reported on family planning, four articles reported on ANC, four articles on labor and delivery, three articles reported on postnatal care, and three articles reported on immunizations. Lastly, seven articles reported on MCH outcomes during the pandemic, as shown in Table 3. The table indicates the themes and subthemes generated from the included papers. In the following section, we describe these in detail.

### 3.3. Scoping Review Themes and Subthemes

#### 3.3.1. Access and Utilization of MNCH Services During the COVID‐19 Pandemic

Nineteen of the 25 reviewed studies reported a decline in maternal, newborn, and child health (MNCH) service utilization, covering family planning, ANC, labor and delivery, postnatal care, immunization, and cervical cancer screening, during the COVID‐19 pandemic, largely due to lockdown restrictions, fear of infection, and stockouts of essential commodities. However, 12 studies demonstrated resilience and partial recovery in service utilization during and postlockdown.

Overall, COVID‐19 significantly disrupted MNCH service utilization across SA, with sharp declines during early lockdowns, especially in family planning, ANC, and immunization. Yet, by late 2020, recovery trends reflected system resilience, supported by adaptation measures and reduced restrictions. Persistent gaps in postnatal care and cervical cancer screening highlight areas needing targeted recovery and resource allocation. The following is a detail for each service.

##### 3.3.1.1. Family Planning

Most studies reported a decline in FP services [21–27]. Pillay et al. [18] found that total FP visits in SA dropped from 96.6 million in 2019 to 81.2 million in 2020, with provincial declines ranging from 8.7% to 31.1%. Adelekan et al. [23] observed a 30% reduction in FP visits during the early lockdown, particularly in April‐May 2020, partly due to stockouts of injectable contraceptives and limited uptake of long‐acting methods. Similar trends were noted in Mpumalanga (21.1% decline) and Free State (1.6%) [18]. Kunle Alabi et al. [25] and Kruger et al. [26] also reported declines in couple years of protection (CYP), dropping by nearly half between February and April 2020.

Despite these declines, some resilience was observed. Adelekan et al. [23] and Siedner et al.​ [28] found an increase in oral contraceptive uptake, rising by 30% in April 2020 compared to 2018‐2019, and a 66% rise in FP visits per clinic/day as lockdown restrictions eased. By late 2020, WHO [27] and Palo et al. [21] noted that FP utilization had returned to near prepandemic levels. However, persistent stockouts and socioeconomic disparities limited consistent access [18, 29, 30].

##### 3.3.1.2. ANC

ANC utilization declined in several provinces [17, 26, 31–34], especially during the first lockdown. Thsehla et al. [31] reported reductions in first ANC bookings in urban and wealthier groups, with the largest declines in Free State (−7.3%), Northern Cape (−5.5%), and Gauteng (−5.1%). Provider shortages, lack of PPE, and fear of infection further constrained access [32, 33]. Mutyambizi et al. [34] observed significant decreases in ANC headcounts from April to December 2020 compared to 2019.

Conversely, some studies showed increased ANC visits, particularly in rural areas, possibly due to population migration during lockdown [24]. Soma‐Pillay et al. [24] reported higher first‐visit numbers in 2020 than in 2019, while Kruger et al. [26] noted that early ANC bookings (< 20 weeks) remained stable at ∼65%. WHO [27] also reported slightly higher ANC contacts in 2020 compared to 2019, reflecting adaptive health system responses.

##### 3.3.1.3. Labor and Delivery

Five studies documented declines in institutional deliveries [17, 18, 26, 31, 35]. Kruger et al. [26] reported a decrease in monthly deliveries during 2021/2022 compared to prepandemic averages, while Thsehla et al. [31] observed reductions in C‐sections in some provinces. Jensen et al. [35] found an 8% drop in hospital births in April 2020, followed by a gradual recovery.

In contrast, some facilities recorded increases. Ahmed et al. [17] noted facility‐specific variations, with deliveries at Steve Biko Academic Hospital rising by up to 40% in April 2020. Pillay et al. [18] reported a national 3.7% rise in facility deliveries, with Mpumalanga (+10.4%) and Limpopo (+8.4%) showing the greatest increases, demonstrating heterogeneous effects across regions.

##### 3.3.1.4. Postnatal Care

Two studies [27, 36] showed reduced postnatal visits, particularly after the pandemic onset. Kelly et al. [36] noted restricted baby check‐ups due to limited appointment slots. WHO [27] reported that postnatal contacts declined after March 2020, following early increases prepandemic. However, Palo et al. [21] and Meherali et al. [37] found that adolescent postnatal visits remained relatively stable, suggesting resilience in specific subpopulations.

##### 3.3.1.5. Immunization

Nine studies reported declines in under‐five immunization [18, 21, 26–28, 31, 35, 38, 39]. Pillay et al. [18] found a 4.3% national reduction in children fully immunized at 1 year, with the steepest drops in April 2020 in Northern Cape (−11.8%) and Eastern Cape (−9.9%). Measles first‐dose coverage fell by ∼30% in April 2020, recovering by June [35]. Siedner et al. [28] observed a 60% reduction in immunization visits during the strict lockdown.

Nonetheless, recovery was evident postlockdown. Kruger et al. [26] documented catch‐up gains, with measles first‐dose coverage improving from 62.8% (April 2020) to 90.6% (2021/2022). Pujolar et al. [39] also noted recovery to near prepandemic levels. However, access disparities persisted, especially among poorer urban families [31].

##### 3.3.1.6. Cervical Cancer Screening

Cervical screening services were the most affected. Kruger et al. [26] reported a drop from an average of 5455 samples/month prepandemic to 3453/month in 2020/2021, with the lowest level in July 2020. No studies indicated recovery or increased screening during the pandemic.

##### 3.3.1.7. Socioeconomic and Behavioral Factors

Socioeconomic disparities influenced MNCH service utilization, with poorer populations and urban households more adversely affected [24, 25]. Fear of contracting COVID‐19, mobility restrictions, and health facility reprioritization toward COVID‐19 care were key barriers [32, 33, 38, 40]. Nevertheless, some evidence suggests that adaptive service delivery and eased restrictions helped restore utilization across multiple MNCH domains by late 2020.

#### 3.3.2. How the Pandemic Affected Delivery of MNCH Services

##### 3.3.2.1. Disruptions of Essential Services

Fifteen out of 25 studies reported on disruptions of essential services in family planning, ANC, labor and delivery, postnatal care, and immunization services.

One of the studies described the disruptions in general for all MNCH services [35]. Lockdowns and lack of safety measures disrupted people’s lives, affecting their utilization of MNCH services in general [35].

##### 3.3.2.2. Family Planning

Five studies reported on disruptions to FP service access [18, 25, 30, 36, 41].

Lockdown contributed to a decline in FP services access, and the largest declines were noted in April and May 2020 relative to the same period in 2019 [18]. Routine visits, baby check‐ups, and long‐acting reversible contraceptives were not prioritized [18]. Kunle Alabi et al. [25, 30, 36] indicate that stockouts of FP supplies limited access to contraceptives. Kunle Alabi et al. [25] indicate that stockouts affected FP services, as there was a decrease of 5%. Pillay et al. [18] indicate that HCWs substituted injectables with oral contraceptives to replace contraceptives that were out of stock. HCWs could not provide quality care, as it was reported that they shouted at clients while clients′ fears of contracting COVID‐19 hindered their access to services [36]. Changes in the availability and provision of health services made it difficult for adolescents to access long‐acting reversible contraceptives despite HCWs reporting that the services were available [36]. On the other hand, adolescents reported that access to services was hindered as they were returned by health workers if they visited the facility without appointments [36]. Adolescent’s health service access declined, which HCWs attributed to barriers faced, such as a lack of transport and external factors. It was reported that FP services were not prioritized as they were not considered essential [36]. Humphries et al. [41] report that adolescent girls and young women (AGYW) and sex workers faced the highest disruptions in FP services by 43% and 40%, respectively. Humphries et al. [41] reported that sex workers were at greater risk of experiencing reduced access to FP services, while migrants were at a lower risk of experiencing reduced access to FP services. At a household level, those who lived in traditional housing compared to a house/flat were also found to be at a greater risk of reduced access to FP services [41].

##### 3.3.2.3. ANC

Five studies reported on disruptions of essential services that occurred to ANC services access [33, 34, 39, 40, 42].

Lockdown measures and social distancing reduced access for pregnant women and families/infants to reach antenatal clinics and primary healthcare [34, 39]. Fear of contracting COVID‐19 was reported as the major reason for not attending the clinic, and treatment by nurses also contributed to women’s missing appointments as scheduled [34, 40]. However, modifications were made to how women accessed services such as creating drive‐through consultations and creating virtual platforms for ANC sessions [42]. It was also noted that due to changes in how women access maternal health services, women discontinued attending ANC services [42]. Prenatal and postnatal care journey was provided informally as women chose informal caregiving because of the situation where they found themselves [40]. The use of public transport hindered women from attending clinics for fear of contracting the virus [33]. Health education sessions were also canceled during the COVID‐19 pandemic, and Anokwuru and Mavis [33] highlight that the majority of antenatal women abstained from attending antenatal visits as scheduled for fear of contracting the disease. Anokwuru and Mavis [33] further indicate that restrictions in movement affected women’s antenatal attendance.

##### 3.3.2.4. Labor and Delivery

Four studies reported on disruptions of essential services that occurred with labor and delivery services access [21, 26, 40, 42].

Disruptions of health services contributed to an increase in neonatal mortality [21]. Prenatal and postnatal care journey was provided informally as women chose informal caregiving not by choice but because of the situation they found themselves in [40, 42]. There was an increase in babies born before arrival (BBAs) as indicated by Kruger et al. [26]. Mohulatsi et al. [42] indicated that women lacked support from their loved ones during the birthing experience as a result of COVID‐19.

##### 3.3.2.5. Postnatal Care

Three studies reported on disruptions of essential services that occurred with postnatal care services on access [22, 36, 40].

Routine visits, baby check‐ups, and long‐acting reversible contraceptives were not prioritized [22]. Prenatal and postnatal care journey was provided informally as women choose informal care giving not by choice but because of the situation where they found themselves [40]. Kelly et al. [36] report that baby check‐ups, although available, were difficult to access as clients could be returned if they did not have an appointment since they were not considered emergencies.

##### 3.3.2.6. Immunizations

Three studies reported on disruptions of essential services that occurred with immunization services on access [33, 38, 39].

Lockdown measures and social distancing reduced access for pregnant women and families/infants to reach antenatal clinics and primary healthcare [38]. Also, disruptions to clinics, canceled clinics, unavailability of vaccines, and staff shortages were reported as hindering access to vaccine uptake and delivery [33, 39]. Fear of COVID‐19 also hindered uptake of vaccines [38].

##### 3.3.2.7. How the COVID‐19 Pandemic Affected Health Outcomes

Seven out of 25 studies reported on health outcomes of maternal deaths, still births, perinatal mortality, and neonatal mortality rate [18, 21, 22, 24–26, 31].

The study reported a 64% increase in maternal deaths in Quarter 4 of fiscal year 2020/21 when compared to the prepandemic rates in 2019/20 [22]. Pattinson et al. [22] further reported 920 and 1273 maternal deaths in fiscal year 2019/20 and 2020/21, respectively. A study by Pattinson et al. [22] reported that nationally maternal deaths and institutional maternal mortality (IMMR) rates increased significantly (*p* ≤ 0.0001) in all provinces except in Northern Cape. The largest percentage increase (> 40%) was in Free State (42%), KwaZulu‐Natal (57%), Mpumalanga (58%), and Western Cape (86%) [22]. Soma‐Pillay et al. [24] indicate that the IMMR increased in all areas after the lockdown from April 2020 to August 2020. Thsehla et al. [31] indicated that maternal deaths in facilities increased by 18% comparing pre‐ and post‐COVID‐19 between April and September 2020. Thsehla et al. [31] further indicate that when the analysis was extended to 2021, the maternal mortality increased to 33%. Pillay et al. [18] found that nationally, 967 maternal deaths were reported from March to December 2020 compared with 788 during the same period in 2019, showing an increase of 179 deaths (22.7%). For neonatal mortality, Pillay et al. [18] reported an increase of 487 (4.8%) in institutional mortality between March 2019 and December 2020. The study reported that the institutional neonatal mortality rate increased from 12.0/1000 live births to 12.2/live births in 2020, with the greatest changes in North West (25.1%), Mpumalanga (10.2%), Gauteng (9.5%), and Western Cape (8.3%) [18]. Palo et al. [21] indicate that a study reported a 47% increase in neonatal in‐facility mortality due to disruptions of health services during COVID‐19. Kunle Alabi et al. [25] report that SA recorded a 3.4% increase in perinatal mortality due to COVID‐19. Kruger et al. [26] report that stillbirths fluctuated with an average number of 98/month (prepandemic), 109/month (2020‐2021), and 98/month (2021‐2022), with spikes noticeable in the first COVID‐19 wave and more sustained peak stretched from second to the third waves (*p* = 0.045; 2020‐2021 vs. prepandemic).

## 4. Discussion

The COVID‐19 pandemic impacted access to MNCH services and delivery of MNCH services. In this review, 25 articles showed the impact the COVID‐19 pandemic had on access to MNCH and delivery of MNCH services. The results and analysis focused on the access and delivery of services as main themes and MCH pillars, namely, family planning, ANC, labor and delivery, postnatal care, under‐five immunization, and cervical cancer screening as subthemes. The articles showed both positive and negative impacts of the COVID‐19 pandemic on access and delivery of MCH services.

First, in this review, we established both a decline and maintained or increased use of FP services; the decline was mainly observed when the lockdown was first initiated in April and with the second lockdown measures in May 2020 [18, 23, 25]. Access to FP is one of the strategies to improve maternal health, and it is cost‐effective as it prevents unintended pregnancies and sexually transmitted diseases, which would help the government save a lot of expenditures [43]. The main reasons for the declines during the lockdown were issues related to stockouts of contraceptives [18]. Facilities also faced an inadequate supply of contraceptives, contributing to lower numbers of clients accessing the services [23]. It is therefore critical to address stockouts even during the emergence of pandemics such as COVID‐19 so that women can access the services.

On a positive note, Palo et al. [21, 27] indicated that there was a reversal in FP utilization as the numbers returned to prepandemic levels toward the end of 2020. This can be attributed to concerted efforts, which the NDoH and several government arms put in place to ensure that essential services are not disturbed. In addition, other studies have shown an increase in some of the FP methods uptake during the pandemic [18, 23, 28, 29]. Pillay et al. [18, 23] indicated that oral contraceptive pills (OCPs) uptake increased and was the most dispensed contraceptive during the lockdown period in April 2020. Hormonal contraceptive uptake also increased, although the data showed a reduction in the monthly average over the past 2 years before COVID‐19 [24]. Siedner et al. [28, 29, 37] reported an increase in FP access, which was more prominent with the easing of lockdown measures. It is interesting to note that access to FP services was reported in these studies [28, 29, 37] as increasing, although some studies, which were performed in Africa, Ethiopia, and Mozambique by Adu et al. [44–46], have reported that fear of contracting disease would prevent women from accessing MNCH services. It is therefore critical to learn from the FP models of service delivery during pandemics to ensure the maintenance of essential services during such public health emergencies.

In addition, a decline in ANC visits was observed in 2020 as compared to 2019, attributed to lockdown measures, adjustments to clinics, a shortage of staff, a lack of PPE, and a fear of contracting COVID‐19 [17, 26, 32–34]. The decline was mainly noted during the months when SA had strict lockdown measures in April and May 2020, compared to the same months in 2019 [17]. Thsehla et al. [31] indicate that the decline in ANC was observed in urban areas rather than rural areas and among wealthy families. This could be due to lockdown measures that were put in place that interfered with people’s lives [17]. The other reason for the decline in ANC visits in urban areas could be that people were migrating to rural areas due to tough socioeconomic situations brought in by COVID‐19 [24]. In addition, it has been established that clients were abstaining from visiting the facilities due to fear of contracting COVID‐19 [33, 47, 48]. ANC is the care that women receive from skilled healthcare professionals when they are pregnant before giving birth and is one of the important pillars for safe motherhood [49–51]. ANC helps reduce maternal and perinatal morbidity and mortality as women are monitored for any complications that come with pregnancy, checked for diseases, and given appropriate treatment and supplements to keep them healthy [52]. Women at increased risk of complications during labor and delivery are identified early during pregnancy, facilitating their referral to appropriate care [51]. It is therefore important during pandemics to ensure that ANC care is maintained and not disrupted. In some studies, it was reported that access to ANC services showed an increase in 2020 and 2021 compared to 2019 [24, 26, 27, 31]. However, it is important to note that the delay in first antenatal visits and the increase in the visits were inconsistent. Soma‐Pillay et al. [24] reported that there were fewer visits in Quarters 2 and 3 despite the increase noted, and that this increase was observed in rural areas rather than urban areas, which might have contributed to migration from urban settings to rural areas. The trend of urban–rural seasonal migrations has been well documented to contribute to access declines in the urban setting [53].

On the part of labor and delivery, the decline in deliveries was observed during the first wave of the COVID‐19 pandemic and some recovery thereafter [18, 26, 31, 33]. In contrast, Ahmed et al. [17] observed that the data were mixed as some months showed either a decline or an increase in facility deliveries. It could be argued that this could be a result of the COVID‐19 measures that were put in place, which could have been affecting people’s ability to reach the facilities until the measures were lifted, contributing to increased numbers. On the other hand, Ahmed et al. [17, 18, 31] while reporting that there was a decrease in facility deliveries also indicated that there was an increase in facility deliveries noted in some provinces. Health facility deliveries are important as skilled HCWs who have knowledge, skills, and appropriate equipment to perform a safe delivery are available and can handle any emergencies on time to save the life of both the mother and the newborn [7, 54]. Availability of skilled attendants in health facility delivery helps reduce maternal and child mortality [55], hence it is important to ensure that labor and delivery services are not interrupted during pandemics.

One study reported that access to 6 days of postnatal care was lower after the pandemic started and for the rest of 2020 [27]. While this study did not indicate the reasons for this, it could be argued that the care‐seeking behavior of pregnant and postnatal mothers as they seek informal care during the pandemic could contribute to this. Dvora et al. [40] indicated that pregnant and postnatal women were seeking informal care because of the situation they found themselves in during the COVID‐19 pandemic.

This review found varied performances in immunization access, for instance, a decline in under‐five full immunization, measles first dose, and measles third dose hexavalent [18, 21, 27, 28, 31, 35]. Jensen et al. [35] indicated that trends for various immunizations were almost the same, although it was for a shorter period. On the other hand, Kruger et al. [26] noted that while there was a sharp decline in measles at the start of the lockdown in April during the Level 5 lockdown, there were subsequent improvements noted with deworming medication as the lockdown eased. These were attributed to misinformation and COVID‐19‐related issues such as service and supply chain disruptions, resource diversion to response efforts, and containment measures that limited immunization service access and availability. Other studies reported an increase in children fully immunized and immunization coverage [26, 39, 56]. These increases have been attributed to the prepandemic strong existing service delivery models and structures [39, 56]. It is therefore important to strengthen the health system delivery structures to ensure resilience during pandemics such as COVID‐19.

Kruger et al. [26] reported that there was a decrease in cervical cancer screening. However, Kruger et al.​ [26] note that changes in the indicator definition “from number of cancer screening performed in women over 30 years of age to also include the number of cervical cancer screening performed for women living with human immunodeficiency virus (HIV) above 20 years of age” caused a discrepancy in the data that were collected. Since this was the only study reporting on cervical cancer screening and given the highlight by the author that the definition changed causing the discrepancy, it can therefore be argued that there is a need to have more information on cervical cancer screening to get a clear picture of how cervical cancer screening was impacted by the COVID ‐19 pandemic.

In addition to focusing on access and demand for health services, in this study, we also looked at the delivery of MNCH services, which were reported to be disrupted during the COVID‐19 pandemic. Lockdown and physical distance affected access and how services were rendered [34, 35, 39]. These measures barred service users from accessing the services in SA and across the country [18, 45]. In addition, due to competing procurement priorities, stockouts of supplies were reported to limit access during the COVID‐19 pandemic [18, 25, 30, 36]. Despite some facilities creating drive‐through consultations and virtual platforms to provide information, women discontinued attending ANC due to these changes that were made [42, 48]. In addition, staff shortages were worsened, which hindered access to uptake and delivery of vaccines and other child health services [32, 35]. This illustrates the negative impact of COVID‐19 on the access and delivery of MCH services.

Literature has shown that if pregnant women do not get appropriate quality care during antenatal, labor and delivery, it results in poor maternal and neonatal outcomes [37]. An increase in maternal deaths was reported by Pattinson et al. [22, 24, 31] for the year 2020/2021 compared to 2019/2020. Pattinson et al. [22] indicate that an increase in IMMR was noted in all provinces except the Northern Cape. Soma‐Pillay et al. [24] noted that IMMR increased after the lockdown period between April and September 2020. This is conquered by Bikwa et al. [57], in a study that was performed in Zimbabwe, who found that there were an increase in adverse maternal and neonatal outcomes during lockdown and higher numbers of maternal deaths in 2020 compared to 2019. Palo et al. [21] reported an increase in neonatal mortality, while Kunle Alabi et al. [25] indicated that SA recorded an increase in perinatal mortality. Kruger et al. [26] also noted that there was a fluctuation in still births with spikes noticeable during the COVID‐19 wave spikes. Therefore, to avoid morbidity and mortality among women and children, there is a need for the government to ensure that ANC services are not disrupted, as some of the contributing factors can be detected early during ANC [50].

### 4.1. Limitations

The process of conducting this scoping review was delayed due to external factors faced by the author such as limited financial resources. By the time the data search for this scoping review was done, few studies had been conducted in SA about the identified topic, which would have an impact on the data gathered, not to be generalized. The articles identified focused only on specific provinces or health facilities and not the whole country, which limited the assessment of the impact on the national scale.

## 5. Conclusion

The review highlights both positive and negative effects of the COVID‐19 pandemic on MNCH services in SA. At the onset of the pandemic, there was a sharp decline in services such as family planning, ANC, labor and delivery, postnatal care, under‐five immunizations, and cervical cancer screening, particularly in April 2020. This drop was largely due to lockdown measures, supply shortages, fear of infection, and changes in service delivery.

Despite these disruptions, some regions demonstrated resilience, with increased visits for ANC, adolescent family planning, and postnatal services. However, service interruptions were associated with a rise in maternal deaths, stillbirths, and perinatal and neonatal mortality.

Encouragingly, several studies reported a rebound in MNCH service utilization over time, especially in family planning, ANC, labor and delivery, and under‐five immunizations. This suggests that certain provincial health systems adapted effectively to the challenges posed by the pandemic.

These insights are vital for guiding policymakers, health managers, and frontline workers in preparing for future public health emergencies. Ensuring continuity of MNCH services during crises must remain a priority. Strengthening health systems and enhancing resilience are essential to protect MCH, even amid disruptions.

## Ethics Statement

Ethical clearance was obtained from the University of the Witwatersrand (approval certificate: #M200706) and Stellenbosch University (#N22/06/068_RECIP_WITS_M200706). Permission to use the public service data was sought from the South African National Department of Health.

## Disclosure

The views and opinions expressed in this article are those of the authors and do not necessarily reflect the official policy or position of any affiliated agency of the authors. All authors read and approved the final manuscript. The opinions expressed are those of the authors and do not necessarily represent the official views of the SAMRC.

## Conflicts of Interest

The authors declare no conflicts of interest.

## Author Contributions

All authors were involved in the development and finalization of this paper. They were all involved from the conception of the topic and writing up of this paper. Delia Chikuse is the guarantor of the manuscript and the corresponding author.

## Funding

This research is part of the bigger research project on the COVID‐19 pandemic and health systems response in South Africa and Malawi. We acknowledge that the RSA part of this study received funding from the South African Medical Research Council (SAMRC) under a Self‐initiated Research (SIR) grant (Grant no. MRC‐48252). The findings from this paper inform the bigger study.

## Supporting Information

Additional supporting information can be found online in the Supporting Information section.

## Supporting information


**Supporting Information 1** All supporting information is included in the manuscript. The PRISMA‐ScR‐Fillable‐Checklist is included in the supporting file.

**Supporting Information 2** Appendix 1: Research protocol.

**Supporting Information 3** Table 2: Atlas.ti Data analysis table.

**Supporting Information 4** Table 3: Included studies of landscape on the effects of the COVID‐19 pandemic on access to MCH services in South Africa: A scoping review.

## Data Availability

Data sharing does not apply to this article as no new data were created or analyzed for this study.
