# Supplementary material for: The Landscape on Access to Maternal and Child Health Services During the COVID‐19 Pandemic in South Africa: A Scoping Review
Source: Interdiscip Perspect Infect Dis. 2026 Jan 29;2026:9065224. doi: 10.1155/ipid/9065224 (PMC12854166; doi:10.1155/ipid/9065224)
Supplement: Supplementary file 2 — Supporting Information 2 Appendix 1: Research protocol. [file IPID-2026-9065224-s002.docx]

Appendix 1: Research Protocol

**Title:** The landscape on access to maternal and child health services during the COVID – 19 pandemic in South Africa: A Scoping Review

**Institution:** University of Stellenbosch

Department of Global Health

Faculty of Health Sciences and Medicine

**Name of Postgraduate Student:** Delia Chikuse

**Contact Email address**: deliachikuse2@gmail.com

**Supervisor:** Dr. Juliet Nyasulu

**Co- Supervisor:** Dr Jeannine Uwimana-Nicol

**Co-Supervisor:** Dr Lynn Hendricks

This is in partial fulfilment of the Masters of Philosophy in Health Systems and Research degree at Stellenbosch University, Cape Town, South Africa, Stellenbosch University

**Background**

Coronavirus disease 2019 (COVID-19) is defined as “illness caused by a novel coronavirus called severe acute respiratory syndrome coronavirus 2 (SARS-CoV-2; formerly called 2019-nCOV).” (Cennimo et al., 2020). According to Jin et al. (2020), COVID-19 was identified in Wuhan, China, in December 2019. Following this, in early March 2020, the World Health Organization (WHO) declared COVID-19 a pandemic (WHO, 2020). The first case in South Africa (SA) was confirmed early in March 2020 (Sekyere et. al., 2020), and in the first quarter of 2023, SA recorded the highest numbers of cumulative COVID-19 cases on the continent, with 4,050 cases (WHO, 2023).

The COVID-19 pandemic affected access to maternal and child health services in many countries. Therefore, this study will focus on determining the evidence available on how COVID-19 affected access to maternal and child health (MCH) services in South Africa. In countries such as Haiti and Sierra Leone, Aranda et al. (2022) observed a decline in antenatal care (ANC) visits, while a decrease in facility-based delivery was observed in Haiti, Liberia, Mexico, and Sierra Leone. The decline in ANC visits and facility-based deliveries was attributed to fear of contracting COVID-19 and lockdown regulations (Aranda et al., 2022). This was conquered by Burger et al. (2021), who found that in South Africa, fear of contracting coronavirus during the COVID-19 pandemic was one of the top reasons people did not seek care. According to the WHO (2020), disruptions in essential services due to the COVID-19 pandemic occurred worldwide. WHO (2020) indicated that the essential services that were disrupted included reproductive, maternal, neonatal, and child health (RMNCH) and nutrition. Pregnant women's fear of contracting COVID-19 was not the only factor hindering access to health facilities (WHO, 2020). Similarly, Mochache et al. (2022) indicated that when facilities were being decontaminated and health workers were sick with COVID-19, service deliveries were affected as facilities were closed. It could be argued that this affected service delivery, as in Pakistan, it is reported that there was a gradual and consistent decline in the utilization of all RMNCH services, whereby family planning decreased by 50.3%, antenatal first visits decreased by 37.5%, and postnatal visits decreased by 37.5% during peak COVID-19 waves (Emmanuel et al., 2022). In South Africa, utilization of RMNCH services such as ANC, family planning, and immunization declined due to lockdown measures and a lack of medical supplies for health care workers, whereby ANC visits and hospital-based deliveries decreased by 17.5% and 28.8%, respectively (Ahmed et al., 2021; Pillay et al., 2021).

The World Health Organization developed the Strategic Preparedness Response Plan (SPRP) in 2021 in order to help countries navigate through the COVID-19 challenges (WHO, 2021). One of the pillars of SPRP aimed at “providing support to countries to develop program-specific strategies to strengthen essential health services and systems,” which included RMNCH. South Africa developed risk-adjusted strategies that limited human interaction to curb the spread of COVID-19 while ensuring that essential services were not affected (Moonasar et al., 2021). However, studies by Ahmed et al. (2021) and Pillay et al. (2021), as indicated above, show that essential services were affected by different challenges during COVID-19. It should be noted that these studies were done in the same year the SPRP was developed; hence, countries including South Africa could have been adjusting to the effects of COVID-19, hence the challenges highlighted.

**Rationale**

Studies in the literature highlight that the COVID-19 pandemic is likely to only reveal its consequences after significant time passes, and literature published before or immediately after policies are implemented may not capture all relevant outcomes (Kotlar et al., 2021). Therefore, this scoping review aims to determine and describe the existing evidence landscape on the effect of COVID-19 on the access and delivery of MCH in South Africa. The findings of this scoping review will contribute to the body of knowledge by providing a landscape of evidence that exists on the effect of COVID-19 on the access and delivery of MCH in South Africa, as well as the research gaps that will inform future research that will potentially inform practice and policy on health system preparedness to improve the provision of MCH services during pandemic outbreaks.

Aim: To determine and describe the existing evidence landscape on the effect of COVID-19 on the access and delivery of MCH services in South Africa.

Objectives:

To determine existing evidence on the effects of the COVID-19 pandemic on access to maternal and child health services

To describe the existing evidence on the effects of the COVID-19 pandemic on the delivery of maternal and child health services

Review question: What is the landscape of evidence that exists on the effect of COVID-19 on the access and delivery of maternal and child health services in South Africa?

**Methodology**

A scoping review will be used to synthesize the literature on studies that have been done on the effects of the COVID-19 pandemic and access to maternal and child health services among women in South Africa. Scoping review uses broader criteria (participants, concept, and context) that avoid a narrow focus and enable a wider range of papers to be considered for inclusion (Lockwood et al., 2019). Munn et al. (2018) indicate that scoping reviews have a broader scope than traditional systematic reviews, with correspondingly more expansive inclusion criteria. Munn et al. (2018) further indicate that scoping reviews will “identify the types of available evidence in a given field.”.

This will map the evidence available on the effects of COVID-19 on access to maternal and child health services that will guide policy formulation on maternal and child health service provision during pandemics in South Africa (Munn et al., 2018).

Participants: In this scoping review, participants will be women and under-five children who access maternal and child health services.

Concept: The purpose of the scoping review is to explore and determine the available evidence on the effects of COVID-19 and access to MNCH services among women and under-five children. These may include but are not limited to, antenatal visits before 20 weeks, under-five immunizations, mother postnatal visits within 6 days after delivery, cervical cancer screening coverage, cervical cancer screening 30 years and older, contraceptive uptake, and contraceptive years dispensed during COVID-19.

Context of interest: This will include studies on the effects of the COVID-19 pandemic in South Africa, whether in private or public hospitals, and services provided at the primary health care level, tertiary level, or community level.

**Search Strategy**

The studies will be identified through search engines such as Google Scholar, PROSPERO, COCHRANE reviews, PubMed, MEDLINE, EBSCOhost, SCOPUS, and gray literature. The search will be limited to articles that were published from January 2019 to March 2023. The first search will be in PubMed using the search terms, and these will be adjusted in other databases to find the available literature. Then the reference list identified in the articles and reports will be searched for additional studies.

The keywords that will be used to search are effects of COVID-19, women, children, maternal health, child health, maternal and child health services, and South Africa.

**Types of Sources**

Articles considered primary studies and secondary studies, such as reviews, grey literature, and newspaper article reports, will be included when conducting searches for the studies.

Data will be captured and then exported to Rayyan software for screening, checking of duplicates, and selection of the final document to be included for review. The search will be conducted by the author with the assistance of Stellenbosch University’s librarian, who is an expert in specialist information.

**Eligibility Criteria**

The eligibility criteria will include studies, reports, and information published online or on websites and other sources, including grey literature on the effects of the COVID-19 pandemic on access to maternal and child health services. Studies published between January 2020 and March 2023, Studies conducted in South Africa and low- and middle-income countries that included South Africa and articles published in English.

**Exclusion Criteria**

The exclusion criteria will be studies that have titles that do not address the effects of the COVID-19 pandemic on access to maternal and child health services among pregnant women. Articles that are not published, Studies that were not conducted in South Africa, LMICs that do not include South Africa, and articles published in other languages than English

**Selection of Studies**

All studies that have been imported into Rayyan software during the search will be independently screened by two reviewers to ascertain eligibility and check for duplicates. The reviewers will check the title and abstract, and then the full text of the study will be retrieved. It is important to have more than one reviewer screen the studies, as this reduces the chance of missing relevant studies (Waffenschmidt et al., 2018). Two reviewers will collect the data independently to avoid selection bias and compare the results later to select the studies to be included in the analysis. If there are any disagreements, a third reviewer will be involved to solve the disagreement, and these will be documented. Data collection forms will be designed, and the forms will include information on the title of the study, participants, author, date of publication, the context of COVID-19 in South Africa, study design, and study results to ensure that the collected studies are selected for analysis (see Supplementary file: Appendix 1).

**Data extraction**

Studies that will be identified with a title and abstract text and with full text will be extracted onto the designed Excel form by the two reviewers. If there are discrepancies between the first two reviews, a third reviewer will be involved to solve the conflict. If studies that were identified during selection do not have a full text, authors will be contacted to provide access, and if not provided, the study will be dropped. If dropped, the details will be documented. The Excel form will be designed for data extraction to collect information on the author, year of publication, country where the study was conducted, aim or purpose, study population, methodology, intervention, outcome measures, and key findings.

The form will be updated whenever necessary during data extraction when the reviewers come across necessary information during charting. According to Peters et al. (2015), it is important to have a trial of the extraction form using two or three studies so that reviewers become familiar with the form. Therefore, the reviewers will have a trial of the form using two of the studies to become familiar with the information that will be extracted (see Supplementary file: Appendix 2).

**Data analysis and presentation**

The analysis of data will be based on the inclusion criteria, and a table will be developed to capture data on the author, year of publication, country where the study was conducted, area of intervention, research methodology, intervention, outcomes, and results or recommendations (see Supplementary file: Appendix 3).

There will be two reviewers to capture the information, and if there are any challenges related to the capture of information for the selected studies among the two reviewers, discussions will be done. If conclusions cannot be reached, opinions will be gathered from third parties, and these will be documented. The themes will be derived from the data that is captured. The studies will be imported into Atlas.ti 22 software for analysis and will be coded. If additional themes are identified in the articles, they will be added to the forms.

Data will be presented in graphs and table forms according to the thematic areas derived from the major themes outlined. The presentation will include a narrative synthesis of what is in the graphs and tables following what has been indicated in the objectives, and the results of the search studies will be presented using a PRISMA flow diagram for scoping review according to Joanna Briggs Institute guidance on scoping review (Peters et al., 2015).

**Ethical Considerations:**

This study is a subset of a bigger study titled Maintaining Essential Services During the COVID-19 Pandemic in Malawi and South Africa: A Call to Strike the Health Systems Balance." The main study, where the main supervisor is a principal investigator, already has ethics approval from Stellenbosch University Health Research and Ethics Committee (HREC) (Appendix 4) and the University of the Witwatersrand (Appendix 5). Therefore, this study requested a waiver since it is a scoping review and there will be no human contact, and the waiver was granted. See approval of the waiver letter in Appendix 6.

Date of Submission: 2023

Registration number: 26716844

**Timeline – September 2022 to December 2023**

| Activity | Sept 22 | Oct 22 | Nov 22 | Dec 22 | Jan 23 | Feb 23 | Mar 23 | Apr 23 | May 23 | Jun  23 | Jul  23 | Aug 23 | Sept 23 | Oct 23 | Nov 23 | Dec23 |
| --- | --- | --- | --- | --- | --- | --- | --- | --- | --- | --- | --- | --- | --- | --- | --- | --- |
| Preparation of Protocol |  |  |  |  |  |  |  |  |  |  |  |  |  |  |  |  |
| Finalisation of protocol |  |  |  |  |  |  |  |  |  |  |  |  |  |  |  |  |
| Submission of Protocol to HREC for Approval |  |  |  |  |  |  |  |  |  |  |  |  |  |  |  |  |
| Registration at Open Access |  |  |  |  |  |  |  |  |  |  |  |  |  |  |  |  |
| Protocol Publication Submission |  |  |  |  |  |  |  |  |  |  |  |  |  |  |  |  |
| Searches for Published and unpublished studies |  |  |  |  |  |  |  |  |  |  |  |  |  |  |  |  |
| Selection of eligible studies |  |  |  |  |  |  |  |  |  |  |  |  |  |  |  |  |
| Data extraction |  |  |  |  |  |  |  |  |  |  |  |  |  |  |  |  |
| Data Analysis |  |  |  |  |  |  |  |  |  |  |  |  |  |  |  |  |
| Report Writing |  |  |  |  |  |  |  |  |  |  |  |  |  |  |  |  |
| Submission of Research Paper |  |  |  |  |  |  |  |  |  |  |  |  |  |  |  |  |

**Budget –**

| **Item** | **Quantity** | **Unit cost** | **Total amount** |
| --- | --- | --- | --- |
| **Information specialist** | **1** | **7500** | **7500** |
| **Research Assistant** | **1** | **7500** | **7500** |
| **Data bundle** | **1** | **3000** | **3000** |
| **Writing retreat for student** | **1** | **7000** | **7000** |
| **Protocol publication** | **1** | **10000** | **10000** |
| **SR review report publication** | **1** | **10000** | **10000** |
|  |  |  |  |
| **Total** |  |  | **45000** |

**Appendix 1 – Study Selection Criteria Form**

|  | Title of Study | Participants | Author | Date of publishing | Context( Covid 19 pandemic in South Africa) | Study Design | Study Results |
| --- | --- | --- | --- | --- | --- | --- | --- |
|  |  |  |  |  |  |  |  |
|  |  |  |  |  |  |  |  |
|  |  |  |  |  |  |  |  |
|  |  |  |  |  |  |  |  |
|  |  |  |  |  |  |  |  |
|  |  |  |  |  |  |  |  |
|  |  |  |  |  |  |  |  |
|  |  |  |  |  |  |  |  |
|  |  |  |  |  |  |  |  |
|  |  |  |  |  |  |  |  |
|  |  |  |  |  |  |  |  |
|  |  |  |  |  |  |  |  |
|  |  |  |  |  |  |  |  |
|  |  |  |  |  |  |  |  |
|  |  |  |  |  |  |  |  |
|  |  |  |  |  |  |  |  |
|  |  |  |  |  |  |  |  |
|  |  |  |  |  |  |  |  |
|  |  |  |  |  |  |  |  |
|  |  |  |  |  |  |  |  |
|  |  |  |  |  |  |  |  |
|  |  |  |  |  |  |  |  |
|  |  |  |  |  |  |  |  |
|  |  |  |  |  |  |  |  |
|  |  |  |  |  |  |  |  |
|  |  |  |  |  |  |  |  |

**Appendix 2 – Data Extraction form**

|  | Author | Year of Publication | Country( where Study was conducted | Aim/  Purpose | Study Population | Methodology | Intervention | Outcome Measure | Key Findings |
| --- | --- | --- | --- | --- | --- | --- | --- | --- | --- |
|  |  |  |  |  |  |  |  |  |  |
|  |  |  |  |  |  |  |  |  |  |
|  |  |  |  |  |  |  |  |  |  |
|  |  |  |  |  |  |  |  |  |  |
|  |  |  |  |  |  |  |  |  |  |
|  |  |  |  |  |  |  |  |  |  |
|  |  |  |  |  |  |  |  |  |  |
|  |  |  |  |  |  |  |  |  |  |
|  |  |  |  |  |  |  |  |  |  |
|  |  |  |  |  |  |  |  |  |  |
|  |  |  |  |  |  |  |  |  |  |
|  |  |  |  |  |  |  |  |  |  |
|  |  |  |  |  |  |  |  |  |  |
|  |  |  |  |  |  |  |  |  |  |
|  |  |  |  |  |  |  |  |  |  |
|  |  |  |  |  |  |  |  |  |  |
|  |  |  |  |  |  |  |  |  |  |
|  |  |  |  |  |  |  |  |  |  |
|  |  |  |  |  |  |  |  |  |  |
|  |  |  |  |  |  |  |  |  |  |
|  |  |  |  |  |  |  |  |  |  |
|  |  |  |  |  |  |  |  |  |  |
|  |  |  |  |  |  |  |  |  |  |
|  |  |  |  |  |  |  |  |  |  |
|  |  |  |  |  |  |  |  |  |  |
|  |  |  |  |  |  |  |  |  |  |
|  |  |  |  |  |  |  |  |  |  |

**Appendix 3 – Data Analysis form**

|  | Author | Year of publication | Country (where study was conducted) | Area of Intervention | Research methodology | Intervention Outcomes | Results/  Recommendation |
| --- | --- | --- | --- | --- | --- | --- | --- |
|  |  |  |  |  |  |  |  |
|  |  |  |  |  |  |  |  |
|  |  |  |  |  |  |  |  |
|  |  |  |  |  |  |  |  |
|  |  |  |  |  |  |  |  |
|  |  |  |  |  |  |  |  |
|  |  |  |  |  |  |  |  |
|  |  |  |  |  |  |  |  |
|  |  |  |  |  |  |  |  |
|  |  |  |  |  |  |  |  |
|  |  |  |  |  |  |  |  |
|  |  |  |  |  |  |  |  |
|  |  |  |  |  |  |  |  |
|  |  |  |  |  |  |  |  |
|  |  |  |  |  |  |  |  |
|  |  |  |  |  |  |  |  |
|  |  |  |  |  |  |  |  |
|  |  |  |  |  |  |  |  |
|  |  |  |  |  |  |  |  |
|  |  |  |  |  |  |  |  |
|  |  |  |  |  |  |  |  |
|  |  |  |  |  |  |  |  |
|  |  |  |  |  |  |  |  |
|  |  |  |  |  |  |  |  |
|  |  |  |  |  |  |  |  |
|  |  |  |  |  |  |  |  |
|  |  |  |  |  |  |  |  |

**References:**

Aranda, Zeus and Binde, Thierry and Tashman, Katherine and Tadikonda, Ananya and Mawindo, Bill and Maweu, Daniel and Boley, Emma Jean and Mphande, Isaac and Dumbuya, Isata and Monta{\~n}o, M. and others. 2022. Disruptions in maternal health service use during the COVID-19 pandemic in 2020: experiences from 37 health facilities in low-income and middle-income countries. *BMJ global health*. 7(1):e007247. DOI: 10.1136/bmjgh-2021-007247.

Burger, Ronelle and Mchenga, M. 2021. Anticipating the impact of COVID-19 pandemic on health inequality in South Africa: early evidence on direct and indirect influences. *Journal of Patient Experience*. 8(June):1–4. Available: https://www.africaportal.org/publications/anticipating-impact-covid-19-pandemic-health-inequality-south-africa-early-evidence-direct-and-indirect-influences/.

Cennimo, D.J., Bergman, S.J. & Olsen, K.M. 2020. Coronavirus Disease 2019 (COVID-19): Practice Essentials, Background, Route of Transmission. Available: https://emedicine.medscape.com/article/2500114-overview.

Emmanuel Sekyere, Narnia Bohler-Muller, Charles Hongoro, and M.M. 2020. The Impact of Covid-19 in South Africa. *Wilson Center*. (April):1–6. Available: https://www.wilsoncenter.org/sites/default/files/media/uploads/documents/The Impact of COVID-19 in South Africa_0.pdf.

Jin, Y.H., Cai, L., Cheng, Z.S., Cheng, H., Deng, T., Fan, Y.P., Fang, C., Huang, D., et al. 2020. A rapid advice guideline for the diagnosis and treatment of 2019 novel coronavirus (2019-nCoV) infected pneumonia (standard version). *Medical Journal of Chinese People’s Liberation Army*. 45(1):1–20. DOI: 10.11855/j.issn.0577-7402.2020.01.01.

Kotlar, Bethany and Gerson, Emily and Petrillo, Sophia and Langer, Ana and Tiemeier, H. 2021. The impact of the COVID-19 pandemic on maternal and perinatal health: a scoping review. *Reproductive Health*. 18(1):1–39. DOI: 10.1186/s12978-021-01070-6.

Lockwood, Craig and Dos Santos, Kelli Borgess and Pap, R. 2019. Practical guidance for knowledge synthesis: Scoping review methods. *Asian nursing research*. 13(5):287–294. DOI: 10.1016/j.anr.2019.11.002.

Mochache, Trufosa and Momanyi, Maureen and Ameda, Ida-Marie and Gohar, Fatima and Ebele, Michael and Odhiambo, Fanuel and Mzozo, Tasiana and Bekele, Hana and Waituru, Mwangi and Nanyunja, M. 2022. Continuity of essential health services in the context of COVID-19: the Eastern and Southern Africa Regional continuity of essential services sub-working group. *The Pan African Medical Journal*. 41(7):1–6. DOI: 10.11604/pamj.supp.2022.41.2.28000.

Moonasar, Devanand and Pillay, Anban and Leonard, Elizabeth and Naidoo, Raveen and Mngemane, Shadrack and Ramkrishna, Wayne and Jamaloodien, Khadija and Lebese, Lebogang and Chetty, Kamy and Bamford, L. and others. 2021. COVID-19: Lessons and experiences from South Africa’s first surge. *BMJ Global Health*. 6(2):e004393. DOI: 10.1136/bmjgh-2020-004393.

Munn, Zachary and Peters, Micah DJ and Stern, Cindy and Tufanaru, Catalin and McArthur, Alexa and Aromataris, E. 2018. Systematic review or scoping review? Guidance for authors when choosing between a systematic or scoping review approach. *BMC medical research methodology*. 18:1--7. Available: https://doi.org/10.1186/s12874-018-0611-x.

Peters, Micah DJ and Godfrey, Christina M and McInerney, Patricia and Soares, Cassia Baldini and Khalil, Hanan and Parker, D. 2015. *The Joanna Briggs Institute reviewers’ manual 2015: methodology for JBI scoping reviews*.

Pillay, Y and Pienaar, S and Barron, P and Zondi, T. 2021. Impact of COVID-19 on routine primary healthcare services in South Africa. *South African Medical Journal*. 111(8):714–719. DOI: 10.7196/SAMJ.2021.V111I8.15786.

Waffenschmidt, Siw and Hausner, Elke and Sieben, Wiebke and Jaschinski, Thomas and Knelangen, Marco and Overesch, I. 2018. Effective study selection using text mining or a single-screening approach: a study protocol. *Systematic Reviews*. 7(1):1–7. DOI: 10.1186/s13643-018-0839-x.

WHO. 2020. *WHO Director-General’s opening remarks at the media briefing on COVID-19 - 11 March 2020*. Available: https://www.who.int/director-general/speeches/detail/who-director-general-s-opening-remarks-at-the-media-briefing-on-covid-19---11-march-2020.

World Health Organization. 2020. *Pulse survey on continuity of essential health services during the COVID-19 pandemic: interim report, 27 August 2020*. Available: https://apps.who.int/iris/bitstream/handle/10665/334048/WHO-2019-nCoV-EHS_continuity-survey-2020.1-eng.pdf.

World Health Organization. 2021. *COVID-19 strategic preparedness and response plan: 1 February 2021 to 31 January 2022*. Geneva. Available: https://apps.who.int/iris/handle/10665/340072.

World Health Organization. 2023. *Corona Virus Disease(COVID - 19 ) dashboard, with vaccination data*.
